# A city-level dataset of heavy metal emissions into the atmosphere across China from 2015–2020

**DOI:** 10.1038/s41597-024-03089-3

**Published:** 2024-02-29

**Authors:** Qi Dong, Yue Li, Xinhua Wei, Le Jiao, Lina Wu, Zexin Dong, Yi An

**Affiliations:** 1 Agro-Environmental Protection Institute, Ministry of Agriculture and Rural Affairs, Tianjin, 300071 China; 2 Xiangtan Experimental Station of Agro-Environmental Protection Institute, Ministry of Agriculture and Rural Affairs, Xiangtan, Hunan 411199 China; 3 College of Computer Science, Nankai University, Tianjin, 300350 China

**Keywords:** Environmental impact, Atmospheric chemistry

## Abstract

The absence of nationwide distribution data regarding heavy metal emissions into the atmosphere poses a significant constraint in environmental research and public health assessment. In response to the critical data deficiency, we have established a dataset covering Cr, Cd, As, and Pb emissions into the atmosphere (HMEAs, unit: ton) across 367 municipalities in China. Initially, we collected HMEAs data and covariates such as industrial emissions, vehicle emissions, meteorological variables, among other ten indicators. Following this, nine machine learning models, including Linear Regression (LR), Ridge, Bayesian Ridge (Bayesian), K-Neighbors Regressor (KNN), MLP Regressor (MLP), Random Forest Regressor (RF), LGBM Regressor (LGBM), Lasso, and ElasticNet, were assessed using coefficient of determination (R^2^), root-mean-square error (RMSE) and Mean Absolute Error (MAE) on the testing dataset. RF and LGBM models were chosen, due to their favorable predictive performance (R^2^: 0.58–0.84, lower RMSE/MAE), confirming their robustness in modelling. This dataset serves as a valuable resource for informing environmental policies, monitoring air quality, conducting environmental assessments, and facilitating academic research.

## Background & Summary

Currently, heavy metal pollution poses a significant threat to both ecological systems and human health. The main sources of heavy metals encompass industrial activities, mining operations, wastewater discharge, and the use of agrochemicals^1–3^. According to Ni *et al*.^4^, 86% of Cr, 77% of Cd, 80% of As, and 94% of Pb in farmland are derived from atmospheric deposition in China, specifically PM10 and PM2.5, characterized by their small size and higher bioavailability^5^. These particles have an increased capacity for dispersion and long-range transport^6,7^, making them prone to transfer to other carriers such as soil, water, and even plant leaves, subsequently leading to the indirect contamination of crops and water bodies. Moreover, PM2.5 and PM10 particles carrying heavy metals, with high toxicity, concealment, persistence, and biological accumulation^8,9^, can penetrate deep into the respiratory system of humans, giving rise to a spectrum of deleterious health effects^10,11^.

However, despite the daily tracking of PM2.5 and PM10 concentration in most of major cities in China since 2013 and various efforts made to generate HMEAs data^12–17^, an assessment of heavy metal emissions into the atmosphere (HMEAs) across the entire country is still infant.

Creating a nationwide dataset for HMEAs is vital for several reasons. First of all, such dataset ensures the assessment on if air quality meets standards and understand its impact on human health, encouraging the implementation of appropriate preventive measures. Secondly, such dataset can be applied to identify pollution sources, therefore benefiting the formulation of effective pollution management strategies^14,18^. For instance, high levels of Pb in the atmosphere normally indicate highly-possibility of the presence of nearby industrial facilities causing Pb pollution^19,20^. This identification can be discerned by investigating the distribution and emissions of these nearby industrial facilities. Crucially, the HMEAs dataset can also be used for scientific research in areas like atmospheric chemistry, meteorology, and environmental science, aiding in the prediction of future air quality and environmental pollution trends^18^.

Nonetheless, the development of such a dataset poses formidable challenges, primarily due to the significant methodological complexities involved in interpolating limited and sparse point data to produce comprehensive large-scale regional datasets^21^. Machine learning (ML), as a powerful tool for uncovering underlying patterns from both voluminous data and limited sample sizes^22–24^, has been increasingly applied to solve such problems because of its cost-efficiency, predictive accuracy, and robustness^22,25^. For example, Lyu *et al*. employed an enhanced Land-Use Regression model to predict the concentration of PM2.5-bound heavy metals in the eastern region of China^21^.

Therefore, this paper aims to complete the city-level national HMEAs dataset from 2015 to 2020 using a non-interpolation-based, machine learning approach. The decision to focus solely on these six years is due to the limited availability of input variable data. To the best of our knowledge, this is the first dataset of its kind at the city-level for China, covering data from 367 cities.

This city-level national HMEAs dataset can be valuable to a wide audience, including researchers, policymakers, and those interested in the subject. It can help evaluate the health risks associated with exposure to toxic metals, establish reference values for regulations, and track changes in pollution levels over time, which is vital for assessing the effectiveness of pollution control efforts and changes in air quality management practices.

## Methods

**Fig. 1:**
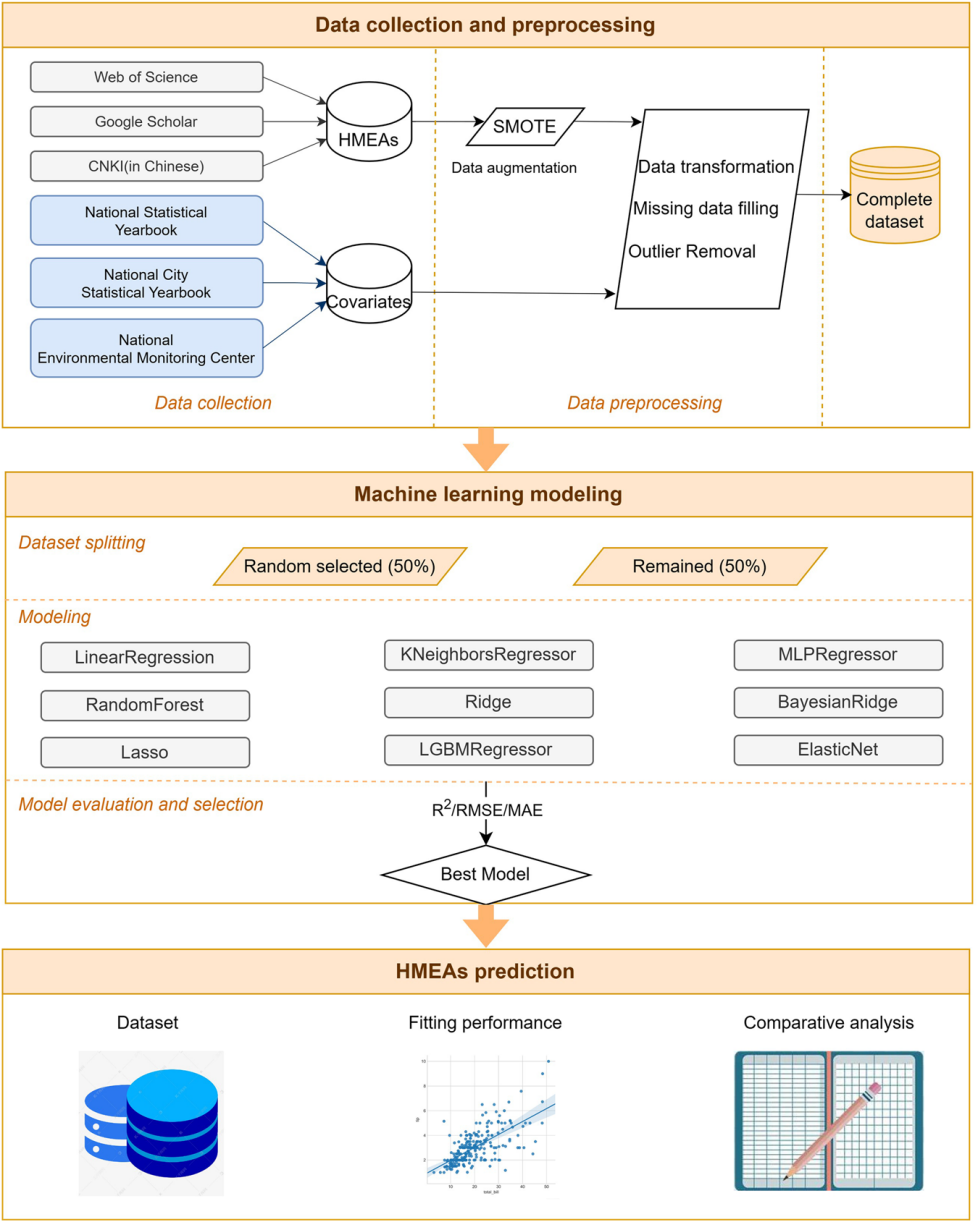
Flowchart diagram of methods to create HMEAs.

An overview of our methods is shown in Fig. 1.

### HMEAs data

To further curb the escalating emissions of heavy metals from fuel combustion and industrial processes, the State Council of the Chinese government officially approved a specific comprehensive prevention plan targeting the five most heavily polluted and toxic HMs (Hg, As, Pb, Cd, and Cr) for the 12th Five-Year Plan (2011–2015). Despite the considerable research on mercury emissions into the atmosphere^26–28^, our study focuses on the other atmospheric heavy metals mentioned above due to the highly volatile and unstable nature of mercury. A comprehensive literature search addressing chromium (Cr), cadmium (Cd), arsenic (As) and lead (Pb) emissions into the atmosphere (abbreviated as CrEA, CdEA, AsEA, and PbEA) from 2000 to 2021 was conducted using Web of science and China National Knowledge Infrastructure (Website: https://www.cnki.net/) to obtain data using the following search terms, where “TS” represents the article theme:

TS = [(PM2.5 OR atmosphere) AND (metal OR metals OR heavy metals OR heavy metal OR Cr OR Cd OR As OR Pb OR chromium OR cadmium OR arsenic OR lead) AND (address: China)] AND (From 2000 to 2021)].

A total of 118175 publications were initially identified. These publications were ranked based on their relevance, with the top 1753 most relevant publications retained. Subsequently, a screening process was implemented by examining the sections of materials and methods to determine the suitability, in total, 208 publications of studies were selected based on the following criteria: (1) clear specification of the sampled particulate matter type, (2) explicit documentation of the sampling site locations, and (3) proper labelling of units for HMEAs data. Because some regions had multiple studies available, spanning different years, we selected those studies that provided comprehensive data for all four target heavy metals. This selection process resulted in a final set of 74 studies. Subsequently, we extracted data from tables and figures using the Web GetData Software (https://getdata.com/). This process yielded a dataset comprising 103 data points for Cr, 98 data points for Cd, 92 data points for As, and 108 data points for Pb.

To calculate the HMEAs (Cr, Cd, As and Pb), the following heuristic formula was employed:

1 $$\begin{array}{l}{\rm{HMEAs}}={\rm{k}}\,\ast \,({\rm{Industrial}}\,{\rm{particulate}}\,{\rm{emissions}})\,\ast \,({\rm{HMEAs}}\,{\rm{concentration}})\\ \,\,\,/({\rm{particulate}}\,{\rm{matter}}\,{\rm{concentration}})\end{array}$$

Where the parameter “Industrial Particulate Emissions” is obtained from the National Statistical Yearbook, and the parameter k represents the unit conversion factor, ensuring that the resulting HMEAs is reported in ton (t). This formula is based on the following rationale: The majority of the HMEAs data found in literatures represent the concentration of heavy metals within particulate matter. Dividing these values by the particulate matter concentration yields the concentration of heavy metals in the atmosphere. Considering that particulate matter is the primary carrier of heavy metals in the atmosphere, and industrial sources contribute approximately 75.4% of the total atmospheric particulate matter emissions (based on the Second National Pollution Source Census Bulletin), we approximately consider industrial particulate matter emissions as atmospheric content. Multiplying this by the concentration of heavy metals in the air results in the heavy metals emissions into the atmosphere.

Particularly noteworthy is that the HMEAs concentration data collected covers the years 2000 to 2021, spanning 22 years. However, the HMEAs dataset constructed in this study is limited to the six years from 2015 to 2020. On one hand, the original HMEAs concentration data is obtained from literature, and its limited volume for the years 2015–2020 raises concerns about the adequacy for subsequent modeling, making it challenging to ensure the generalization capability of the models. On the other hand, the input variables for prediction, such as industrial pollutant emissions and vehicular emissions, are primarily sourced from national statistical yearbooks, with data available for only a few provinces before 2015, and most cities lack data. For these reasons, we utilized data from 2000 to 2021 for modeling and testing model performance, and employed the established model to predict HMEAs data for the years 2015 to 2020.

### Environmental covariates

In this study, environmental covariates, such as industrial emission and Meteorological factors, were chosen based on existing literatures^21,29^, these covariates play crucial roles in shaping air quality cand, consequently, the presence of heavy metals in particulate matter. Considering data integrity concerns, the covariate data utilized for modeling are all based on the data from the year 2015. Here, the detailed rationale for selecting these covariates and their data source are presented as follows:

#### Industrial Pollutants

Industrial emissions, including sulfur dioxide (indSO2) and nitrogen oxides (indNOx), are significant contributors to HMEAs^9,30^. These emissions can serve as oxidizing agents in the atmosphere, reacting with heavy metal compounds and likely transforming them into more mobile and readily dispersible forms^31^, which remarkably influence HMEAs. The data representing industrial pollutants emissions, were acquired from the National Statistical Yearbook.

In the absence of city-level data for indNOx in 2015 and 2016, available only at the provincial level, we employed an estimation method based on the data from 2017 to 2020. The estimation procedure is as follows: To complete the data for the years 2017–2020, we applied linear temporal interpolation to fill in missing values for cities with incomplete data for specific years. At this point, it’s worth mentioning that the missing value filling method used here involves linear interpolation, but the subsequent prediction processes utilize non-linear machine learning methods. We observed that the proportion of indNOx emissions from each city to the corresponding provincial emissions in different years was relatively consistent, with most fluctuations hovering around 10%. Therefore, we calculated the indNOx emission data for the years 2015–2016 based on the provincial emissions and the average city-to-province ratio of emissions from 2017 to 2020.

The emission sources of heavy metals vary significantly across different regions. The uniform adoption of industrial sulfur dioxide and industrial nitrogen oxides as emission sources in this study is justified for the following reasons: First, the study covers a broad scope, spanning the national and municipal levels. Unlike smaller regions where pollution sources and emissions are well-defined, the industrial categories for each municipality are highly complex, making it challenging to ascertain emission quantities for the industrial sources across all 367 municipalities in China. Second, currently available data from Chinese government departments such as the Ministry of Ecology and Environment and the Ministry of Energy only provide total emissions of industrial sulfur dioxide and nitrogen oxides without industry-specific breakdowns.

#### Vehicle Emissions

Vehicle emissions are a major source of nitrogen oxides (carNO_x_) and particulate matter (carSmoke). These emissions can interact with heavy metals in the atmosphere, potentially increasing the overall load of heavy metals^32^. For example, during the operation of vehicles, brake and tire wear can cause the release of heavy metals like Cr and Pb^19^, which can contribute to HMEAs, especially in urban areas with high vehicular traffic. Additionally, the combustion of fuel in vehicles can release emissions containing heavy metals such as As and Pb^33,34^, which may subsequently be absorbed by airborne particulate matter. Data on vehicle emissions, including nitrogen oxides (NOx) and particulate matter, was obtained from both the National Statistical Yearbook and the National City Statistical Yearbook, but only provincial data on vehicle emissions was available. Considering the city-level vehicle emissions were strongly correlated with the number of motor vehicles, the following formula Considering the city-level vehicle emissions were strongly correlated with the number of motor vehicles, the following formula was utilized:

2 $$\begin{array}{l}{\rm{City}}\,{\rm{vehicle}}\,{\rm{emissions}}=({\rm{Provincial}}\,{\rm{vehicle}}\,{\rm{emissions}})\,\ast \,\,\,\,\,\,\\ ({\rm{City}}\,{\rm{vehicle}}\,{\rm{count}}/{\rm{Provincial}}\,{\rm{vehicle}}\,{\rm{count}})\end{array}$$

Since the period from 2015 to 2020 marked the initial stages of the development of new energy vehicles, accounting for a relatively small proportion, ranging from 1.3% to 1.75% of the total number of motor vehicles (data derived from Ministry of Public Security), this study did not take into account the emissions of heavy metals from new energy vehicles.

#### Population

Human activities, including industrial processes and transportation, exhibit a connection with the heavy metals emissions into the atmosphere^35^. The size and density of the population in a given area can affect the local concentration of heavy metals in the atmosphere, consequently affecting the HMEAs, as regions such as Henan, Shandong, and Anhui, with more extensive human activity often experience higher emissions^21^. Population data were retrieved from the National Statistical Yearbook.

#### Meteorological covariates

Meteorological factors can influence the dispersion, transport, and deposition of particulate matter^36,37^, have a strong effect of HMEAs. For instance, due to the scavenging effects on particulate matter by wet deposition, precipitation was negatively correlated with particulate matter concentration, evidently impacting HMEAs^38^. This is attributed to the varying characteristics of heavy metals in the atmosphere, including size distributions, temporal variations, and their relationships with meteorological parameters, these variations contribute to their complex health risks^39^. Meteorological data, including temperature, humidity, sunlight duration, wind speed, and precipitation, were obtained from the China National Environmental Monitoring Center (http://www.cnemc.cn/).

### Data preprocessing

The existing dataset encountered certain challenges characterized by a limited sample size, substantial differences in magnitude between various parameters, and the presence of outliers—extremely large and small values that can significantly contribute to high errors. To address these challenges, data preprocessing was conducted, involving the utilization of the Synthetic Minority Over-sampling Technique (SMOTE) method, which has proven effective in balancing and augmenting data when dealing with limited samples Table 1.

**Table 1:** Descriptive Statistics of CrEA, CdEA, AsEA, PbEA, indSO2 (industrial sulfur dioxide), indNOx (industrial nitrogen oxides), carNOx (vehicle emissions of nitrogen oxides), carSmoke (vehicle emissions of particulate matter), pop (population), temp (temperature), rh (humidity), sd (sunlight duration), wsp (wind speed) and preci (precipitation).

	count	mean	std	min	25%	50%	75%	max	unit
**CrEA**	97	30.35	98.68	0.04	2.33	5.05	18.98	692.98	ton
**CdEA**	90	8.13	32.38	0.01	0.54	1.55	3.23	288.59	ton
**AsEA**	89	24.84	92.74	0.25	2.57	7.12	15.56	865.77	ton
**PbEA**	103	162.61	787.37	0.07	12.05	35.31	79.44	7787.34	ton
**indSO2**	367	41204.61	41303.03	1.36	12852.41	32204	57563.5	426800	ton
**indNOx**	367	30898.21	32357.35	3.73	9165.49	21750.44	41659.84	263378.75	ton
**carNOx**	367	18531.42	20697.94	10	5745.03	11866.86	23275.12	178819.36	ton
**carSmoke**	367	1745.56	1835.67	1.23	531.27	1106.64	2305.74	12601.5	ton
**pop**	367	374.92	345.16	0.13	148.12	295	489.95	3070	10,000 people
**temp**	367	14.51	5.81	−0.54	10.15	15.24	17.77	26.62	°C
**rh**	367	69.09	11.42	35.08	60.76	71.56	79.26	82.84	%
**sd**	367	2009.18	549.8	883.91	1571	2030.53	2418.73	3277.38	hour
**wsp**	367	4.87	1.01	2.88	4.22	4.81	5.45	8.33	m/s
**preci**	367	1146.93	665.02	45.48	573.3	1085.83	1682.51	2836.31	mm

#### Data augmentation

**Fig. 2:**
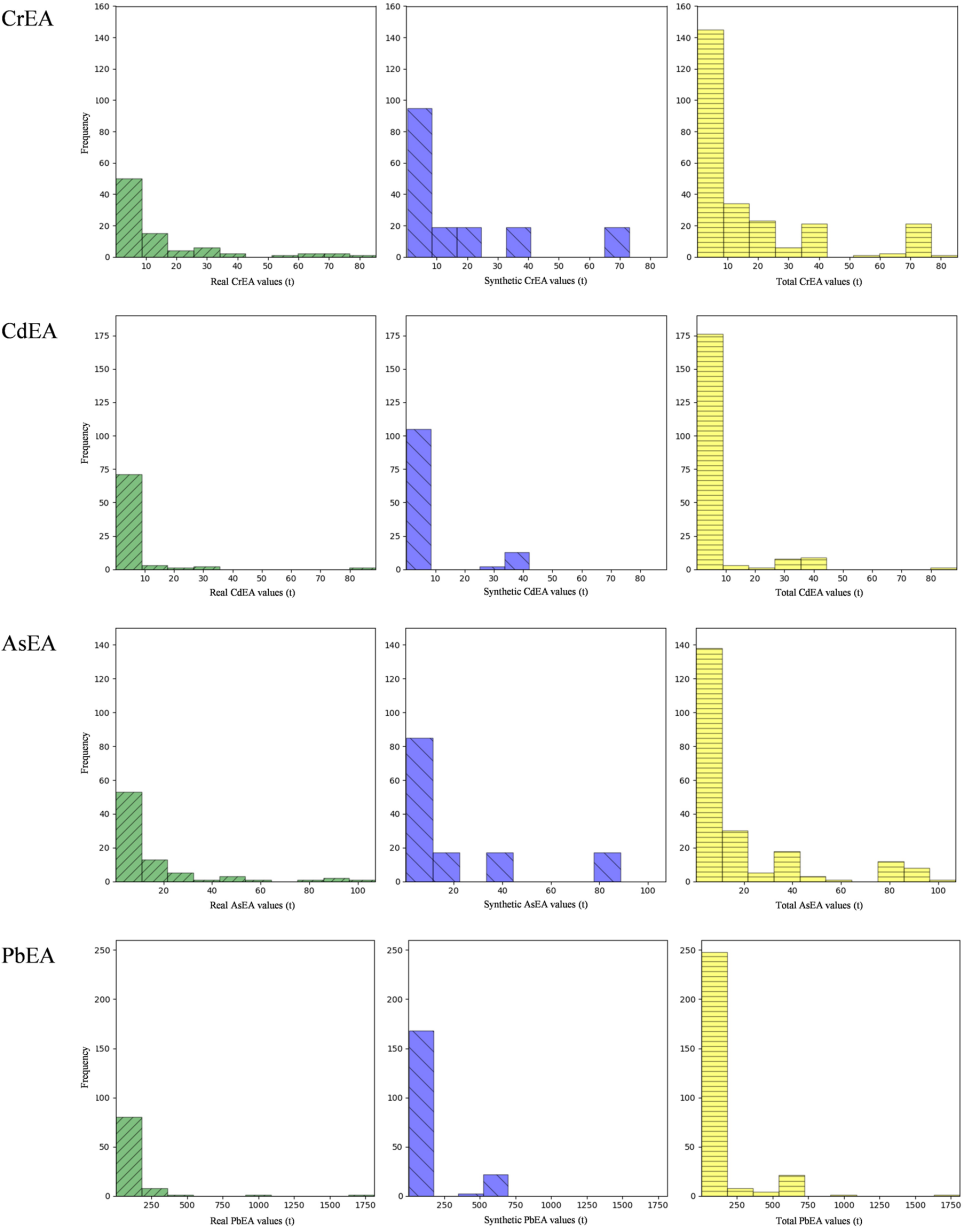
Distribution charts of original data (left) for CrEA/CdEA/AsEA/PbEA, augmented data (center) by SMOTE, and the combined dataset (right).

In this study, we applied SMOTE to expand the dataset, resulting in increased data points for HMEAs, specifically 264 for CrEA, 199 for CdEA, 217 for AsEA, and 285 for PbEA. As demonstrated in Fig. 2, the augmentation process effectively rectified the data distribution, particularly by supplementing the initial dataset with additional data points for the rare, extremely high values, thereby enhancing data balance. A t-test was conducted on the synthetic data generated by SMOTE and the real data (results in Table 2), revealing non-significant differences between the synthetic and real data. This suggests the credibility of the synthetic data.

**Table 2:** The t-test results for synthetic data generated using SMOTE and real data.

HMEAs	P values	CI 95%
CrEA	0.106	−9.49, 0.93
CdEA	0.176	−5.52, 1.02
AsEA	0.159	−11.37, 1.88
PbEA	0.442	−74.6, 32.74

#### Data Transformation

With the purpose of achieving a more favorable approximation to a normal distribution, a natural logarithm transformation was applied to CrEA, CdEA, AsEA, PbEA, indSO2, indNOx, carNOx, carSmoke, sd, wsp, and preci. Subsequently, outliers were removed from each column using a 3-fold standard deviation criterion.

### Machine learning modeling

#### Dataset Splitting

In our data partitioning strategy, we employed 10-fold cross-validation to rigorously assess the performance of the ML models. The dataset comprises both real data and synthetic data generated using SMOTE. For the training set, 50% of the real data was thoughtfully combined with 50% of the synthetically generated data, while the remaining 50% of the real data was reserved for the test set. This process was repeated ten times, with each iteration using a distinct data partition. To emphasize, the test evaluations were conducted exclusively on the entirely real data subset. This approach mitigates the risk of overfitting to the training data, and allows us to evaluate the model based on real data’s characteristics, as the synthetic data was deemed unsuitable for testing.

#### Model Selection

To identify the most suitable model for predicting HMEAs, we evaluated nine machine learning models. Including a variety of machine learning models serves the purpose of exploring different approaches and capturing the diverse patterns present in the data. Among the chosen models, some are linear, while others are non-linear.

Linear Models: including Linear Regression (LR), Ridge, BayesianRidge (Bayesian), Lasso, ElasticNet, these models assume a linear relationship between input features and the target variable. Despite their simplicity, using multiple linear models allows for capturing different aspects of the linear relationship and accounting for potential collinearity issues.

Non-linear Models: including KNeighborsRegressor (KNN), MLPRegressor (MLP), RandomForestRegressor (RF), LGBMRegressor (LGBM), these models are capable of capturing non-linear relationships in the data. KNN relies on local patterns, MLP is a neural network capable of handling complex non-linearities, and RF and LGBM are ensemble methods effective in capturing intricate relationships and feature importance.

The application of multiple linear models is motivated by the desire to investigate different facets of linear relationships and potential collinearity challenges. Additionally, this approach provides a comparison against non-linear models to assess whether the data exhibits significant non-linearities that the linear models may not capture effectively. Parameter design can be provided upon request. The coefficient of determination (R^2^), root-mean-square error (RMSE) and Mean Absolute Error (MAE) on the testing dataset were utilized to compare the prediction performance. R^2^, RMSE and MAE values were calculated using Eqs. 3, 4 and 5, respectively.

3 $${{\rm{R}}}^{2}=\frac{{\sum }_{{\rm{i}}=1}^{{\rm{n}}}{({\widehat{{\rm{y}}}}_{{\rm{i}}}-\bar{\mathrm{y}})}^{2}}{{\sum }_{{\rm{i}}=1}^{{\rm{n}}}{({{\rm{y}}}_{{\rm{i}}}-\bar{\mathrm{y}})}^{2}}$$

4 $${\rm{RMSE}}=\sqrt{\left(\frac{{\sum }_{{\rm{i}}=1}^{{\rm{n}}}{({\widehat{{\rm{y}}}}_{{\rm{i}}}-{{\rm{y}}}_{{\rm{i}}})}^{2}}{{\rm{n}}}\right)}$$

5 $${\rm{MAE}}=\frac{1}{{\rm{n}}}\mathop{\sum }\limits_{{\rm{i}}=1}^{{\rm{n}}}| {\widehat{{\rm{y}}}}_{{\rm{i}}}-{{\rm{y}}}_{{\rm{i}}}| $$

Where ${\widehat{{\rm{y}}}}_{{\rm{i}}}$, y_i_ and $\bar{\mathrm{y}}$ represent the predicted values, observations and average observations, respectively. Models with high R^2^ values and low RMSE and MAE will be selected.

#### Model uncertainty

We calculated the probability of coverage for prediction intervals (PICP). This probability represents the percentage of samples falling within the boundaries of a prediction interval, given a specific level of confidence. If the uncertainty estimates are appropriately determined, the PICP values should approximate 0.90.

### HMEAs prediction creation

Once the models were trained on the training dataset, we utilized the trained models to predict HMEAs for the test dataset. The predictions, initially provided in logarithmic form, were transformed into their original content values. We then assessed the model’s performance by calculating R^2^, RMSE, and MAE between the predicted and actual values. Subsequently, this approach provides a more accurate assessment of the model’s performance, helping to prevent overly optimistic results and ensuring that the model’s predictions are in closer agreement with real observations.

## Data Records

The dataset of HMEAs is available on figshare with a doi of 10.6084/m9.figshare.24762513.v4^40^.

Specifically, the dataset encompasses HMEAs data spanning the time window from 2000 to 2021 at the city level. The files comprise six distinct datasets: CrEA_predictions, CdEA_predictions, AsEA_predictions, PbEA_predictions, HMEAs_data, and Environmental_covariates_data. Each of the first four tables contains eight columns. The first and second columns denote the province and the city, respectively, while columns 3–8 correspond to data for the years 2000–2021, measured in tons.

The HMEAs_data file specifies the sources and references for all original data in the manuscript, providing a comprehensive list of detailed original HMEAs data.

The Environmental_covariates_data table consists of 15 sheets (Table 3), each dedicated to the raw data used in calculating 10 environmental covariates based on the Methods above. These data were obtained from publicly available statistical yearbooks, meteorological monitoring stations, and other sources on the Chinese government website. The raw data, sourced from these platforms, underwent necessary conversions before being incorporated as input data into the model.

**Table 3:** Sheets included in the Environmental_covariates_data.xlsx table.

sheet	explanation	column_name	columns_number
indSmoke	Industrial emissions of particulate matter at the city level	province,city, 2015, 2016, 2017, 2018, 2019, 2020, unit	368
indNOx	Industrial nitrogen oxides emission at the city level	province,city, 2015, 2016, 2017, 2018, 2019, 2020, unit	368
carNOx_province	Vehicle nitrogen oxides emission at the province level	province,2015, 2016, 2017, 2018, 2019, 2020, unit	32
carSmoke_province	Vehicle emissions of particulate matter at the province level	province,2015, 2016, 2017, 2018, 2019, 2020, unit	32
Motor_vehicle_quantity_province	Number of motor vehicles at the province level	province,2015, 2016, 2017, 2018, 2019, 2020, unit	32
Motor_vehicle_quantity_city	Number of motor vehicles at the city level	province,city, 2015, 2016, 2017, 2018, 2019, 2020, unit	368
indNOx_province	Industrial nitrogen oxides emission at the province level	province,2015, 2016, 2017, 2018, 2019, 2020, unit	32
indSO2	Industrial sulfur dioxide emission at the city level	province,city, 2015, 2016, 2017, 2018, 2019, 2020, unit	368
indSO2_province	Industrial sulfur dioxide emission at the province level	province,2015, 2016, 2017, 2018, 2019, 2020, unit	32
pop	Population	province,city, 2015, 2016, 2017, 2018, 2019, 2020, unit	368
temp	Temperature	province,city, 2015, 2016, 2017, 2018, 2019, 2020, unit	368
rh	Humidity	province,city, 2015, 2016, 2017, 2018, 2019, 2020, unit	368
sd	Sunlight duration	province,city, 2015, 2016, 2017, 2018, 2019, 2020, unit	368
wsp	Wind speed	province,city, 2015, 2016, 2017, 2018, 2019, 2020, unit	368
preci	Precipitation	province,city, 2015, 2016, 2017, 2018, 2019, 2020, unit	368

On Github is available the code in Python language to reproduce the HMEAs computation starting from the raw data. In the main folder “Code” there are four sub-folders named “CrEA_code”, “CdEA_code”, “AsEA_code”, and “PbEA_code”, containing the scripts used for the HMEAs computation.

## Technical Validation

### Model performance

The results, averaged over 10-fold cross-validation on the training and testing dataset for R^2^, RMSE, and MAE, are presented in Table 4. All models exhibit lower performance metrics on the excluded testing dataset compared to their training counterparts (10-fold CV). This difference is primarily due to the testing data not being involved in model development and the inherent variability introduced by the random assignment of monitoring sites to the testing set. Among these models, both RF and LGBM consistently exhibited significantly higher R^2^ values and lower RMSE and MAE scores than other models. Specifically, for CrEA, the LGBM model demonstrated superior performance with an R^2^ value of 0.84 (compared to 0.76 for RF), accompanied by lower RMSE and MAE, showing a reduction of 15%-20%. However, a different trend was observed for CdEA, AsEA, and PbEA, where the RF model exhibited top performance, yielding the highest R^2^ values: 0.58 for CdEA (0.41, the second-best result from LGBM), 0.73 for AsEA (0.68, the second-best result from LGBM), and 0.70 for PbEA (0.61, the second-best result from LGBM). Furthermore, the RF model achieved lower RMSE and MAE values by 7%-65% for these three HMEAs. Consequently, based on a comprehensive evaluation and superior performance, the LGBM model was chosen for CrEA, while the RF model was selected for CdEA, AsEA, and PbEA.

**Table 4:** R^2^, RMSE and MAE values for the 10-fold cross-validation testing set (50% of the real data), the units of RMSE and MAE were tons.

HMEAs	metrics	RF	LGBM	LR	Ridge	KNN	BR	Lasso	ElasticNet	MLP
**CrEA**	R^2^_train	0.859	0.886	−0.06	−0.063	0.121	−0.087	−0.209	−0.209	−4.70E + 18
	RMSE_train	8.107	7.297	22.177	22.023	20.335	22.535	23.601	23.581	1.50E + 10
	MAE_train	3.784	3.498	13.397	13.15	11.607	13.314	14.146	14.08	4.80E + 09
	R^2^_test	0.763	0.842	−0.075	−0.069	0.022	−0.051	−0.104	−0.067	−1.30E + 19
	RMSE_test	8.304	6.92	18.715	19.846	16.534	17.714	19.403	18.957	1.60E + 10
	MAE_test	4.084	3.562	10.874	11.769	9.724	10.546	11.265	11.108	5.20E + 09
**CdEA**	R^2^_train	0.776	0.71	−0.018	−0.022	0.08	−0.035	−0.124	−0.118	−4.20E + 27
	RMSE_train	5.673	6.23	11.691	11.736	10.894	11.273	12.273	11.908	2.60E + 14
	MAE_train	1.824	2.049	5.032	5.027	4.625	4.879	5.2	5.112	3.20E + 13
	R^2^_test	0.584	0.411	−0.696	−0.06	−0.026	−0.072	−0.078	−0.055	−3.10E + 25
	RMSE_test	4.392	7.272	9.978	9.707	11.151	12.758	9.884	11.614	1.90E + 13
	MAE_test	1.384	2.066	3.85	3.603	4.249	4.19	3.476	3.927	3.70E + 12
**AsEA**	R^2^_train	0.898	0.856	0.103	0.091	0.242	0.079	−0.076	−0.064	−6.30E + 48
	RMSE_train	8.341	9.856	24.435	25.054	22.622	24.989	27.219	27.043	2.40E + 25
	MAE_train	3.8	4.131	13.641	13.879	12.186	13.867	14.941	14.923	2.50E + 24
	R^2^_test	0.726	0.68	−0.027	0.072	0.083	0.031	−0.035	−0.038	−2.60E + 44
	RMSE_test	10.665	11.877	23.108	18.969	20.354	20.763	19.637	19.6	9.50E + 22
	MAE_test	4.704	5.039	12.702	10.977	11.19	11.137	10.679	10.729	2.50E + 22
**PbEA**	R^2^_train	0.815	0.873	−0.002	−0.008	0.256	−0.022	−0.119	−0.105	−3.50E + 00
	RMSE_train	89.402	72.968	201.37	205.059	171.416	197.746	209.119	209.425	3.20E + 02
	MAE_train	28.219	22.549	93.281	92.812	76.367	90.64	95.742	95.275	1.60E + 02
	R^2^_test	0.696	0.613	−0.414	−0.531	−0.251	−0.229	−0.061	−0.096	−4.80E + 00
	RMSE_test	92.416	106.504	191.131	186.071	189.138	232.47	221.016	198.945	2.80E + 02
	MAE_test	28.879	34.582	75.582	76.165	73.984	86.106	79.895	76.943	1.30E + 02

**Fig. 3:**
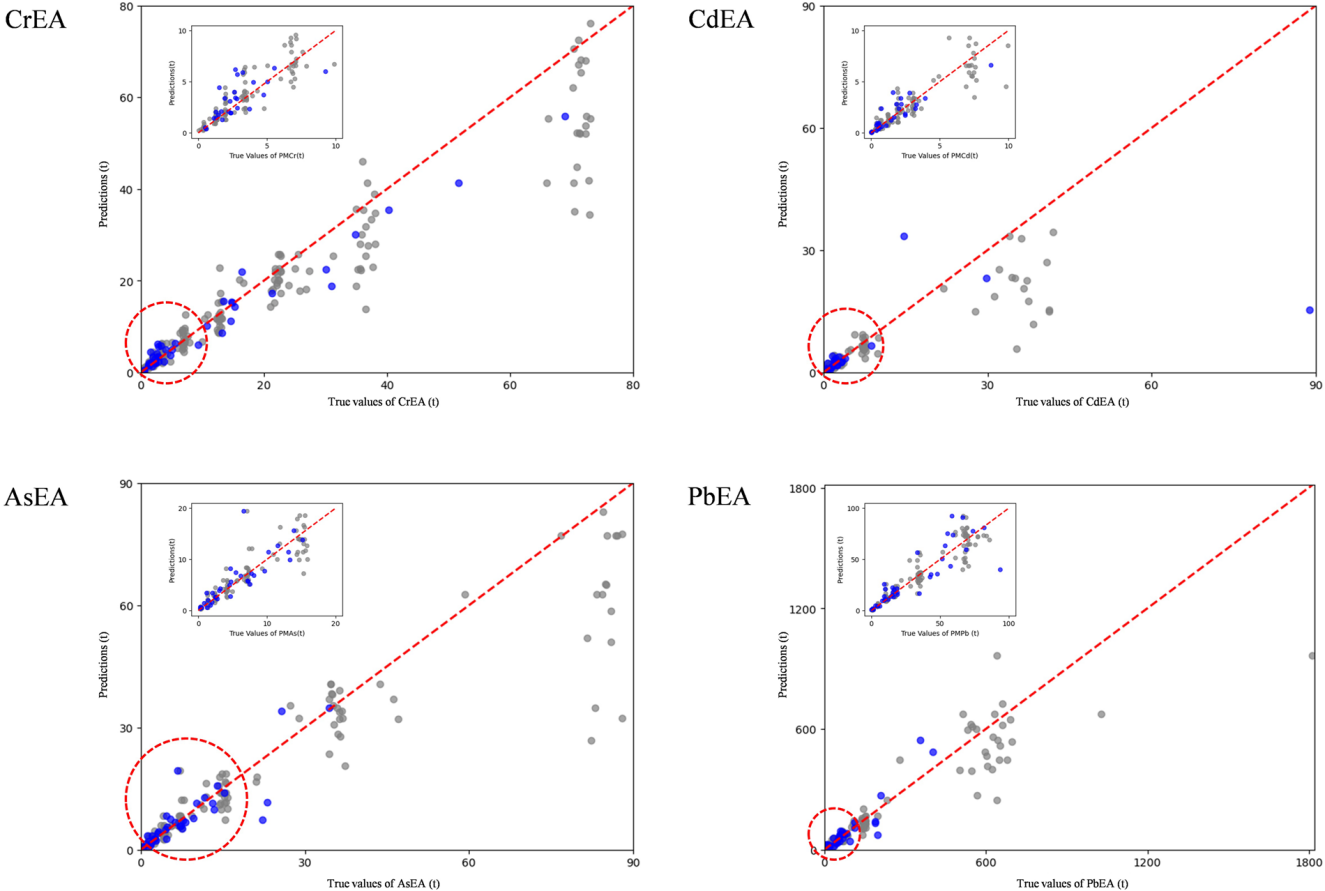
Scatter plot of predicted values versus actual values in training (gray) and testing set (blue). Red circles indicate magnified regions.

The scatter plots depicted values predicted by RF and LGBM versus observed values in Fig. 3. It’s noteworthy that the PbEA dataset exhibits an exceptionally wide numerical range, spanning from 0 to 1800, while the data spans for the other three HMEAs reach a maximum of only 90. Additionally, there are very few values in the PbEA dataset exceeding 700t (only 2), and these two values may be outliers. The models perform well on lower numerical values, encompassing both the training and testing sets, with AsEA standing out. Specifically, the fitted R^2^ for AsEA predictions versus actual values in the testing sets reaches as high as 0.87, with the R^2^ remaining at 0.8 when AsEA values are below 20t. Additionally, CrEA, CdEA, and PbEA demonstrate satisfactory performance in the testing set within the ranges of 0-10t, 0-10t, and 0-100t, respectively, with R^2^ values ranging from 0.44 to 0.72. A consistent trend is observed across all models, indicating an inclination to underestimate HMEAs values beyond the specified numerical ranges. This tendency probably partly stems from the scarcity of high values in the training set compared to low values, suggesting a potential limitation in capturing extreme values during model training. Other factors, such as hyperparameter settings or the unique distribution of data within the context of modeling, may also contribute.

### Predictions assessment

#### Comparative analysis of point data

After predicting HMEAs data for 367 cities nationwide from 2015 to 2020 using the selected model, we compared these predictions with the actual data found in literatures, as shown in Table 5. In comparison, the fitting R^2^ is higher for AsEA, CdEA, and PbEA than that for CrEA, particularly for PbEA, with an average R^2^ value as high as 0.83, and the R^2^ remains consistently above 0.7 for all years, despite the high RMSE and MAE in 2017 and 2018. In general, according to the RMSE and MAE, the order of errors from smallest to largest is: CdEA, CrEA, AsEA, and PbEA, indicating that CdEA values are likely the most accurate. Due to the larger data volume for the four HMEAs and the model being trained based on covariate data from 2015, the fitting R^2^ is relatively higher in this year. Some individual years have R^2^ values exceeding 0.8, such as the 2016 (1.00) and 2020 (0.98) for AsEA, possibly due to the small data size for these two, with only 5 and 3 data points, respectively. Transferring the model trained on 2015 data to other years resulted in a decrease in performance, but overall, it remains acceptable.

**Table 5:** Comparative analysis of model predictions and experimentally measured values recorded in literatures, the units of RMSE and MAE were tons.

HMEAs	Metrics	2015	2016	2017	2018	2019	2020	Average
**CrEA**	n	25	6	7	13	11	4	
	R^2^	0.94	0.33	0.74	0.52	0.35	0.76	0.61
	RMSE	3.49	5.34	1.54	1.87	2.15	6.14	3.42
	MAE	1.91	3.33	1.36	1.47	1.61	4.01	2.28
**CdEA**	n	15	6	12	16	8	4	
	R^2^	0.9	0.88	0.51	0.92	0.39	0.82	0.74
	RMSE	1.36	4.68	1.1	13.93	0.43	0.18	3.61
	MAE	0.63	2.64	0.77	5.47	0.38	0.15	1.67
**AsEA**	n	14	5	9	16	9	3	
	R^2^	0.77	1	0.46	0.64	0.36	0.98	0.7
	RMSE	6.22	11.39	3.15	26.51	2.53	0.68	8.41
	MAE	3.55	5.54	2.64	14.73	2.22	0.5	4.86
**PbEA**	n	12	6	14	18	8	3	
	R^2^	0.98	0.72	0.75	0.74	0.94	0.84	0.83
	RMSE	8.8	12.86	53.92	68.17	7.21	5.03	26
	MAE	5.75	10.09	34.04	36.87	5.83	4.66	16.21

#### Comparative analysis of annual totals

**Fig. 4:**
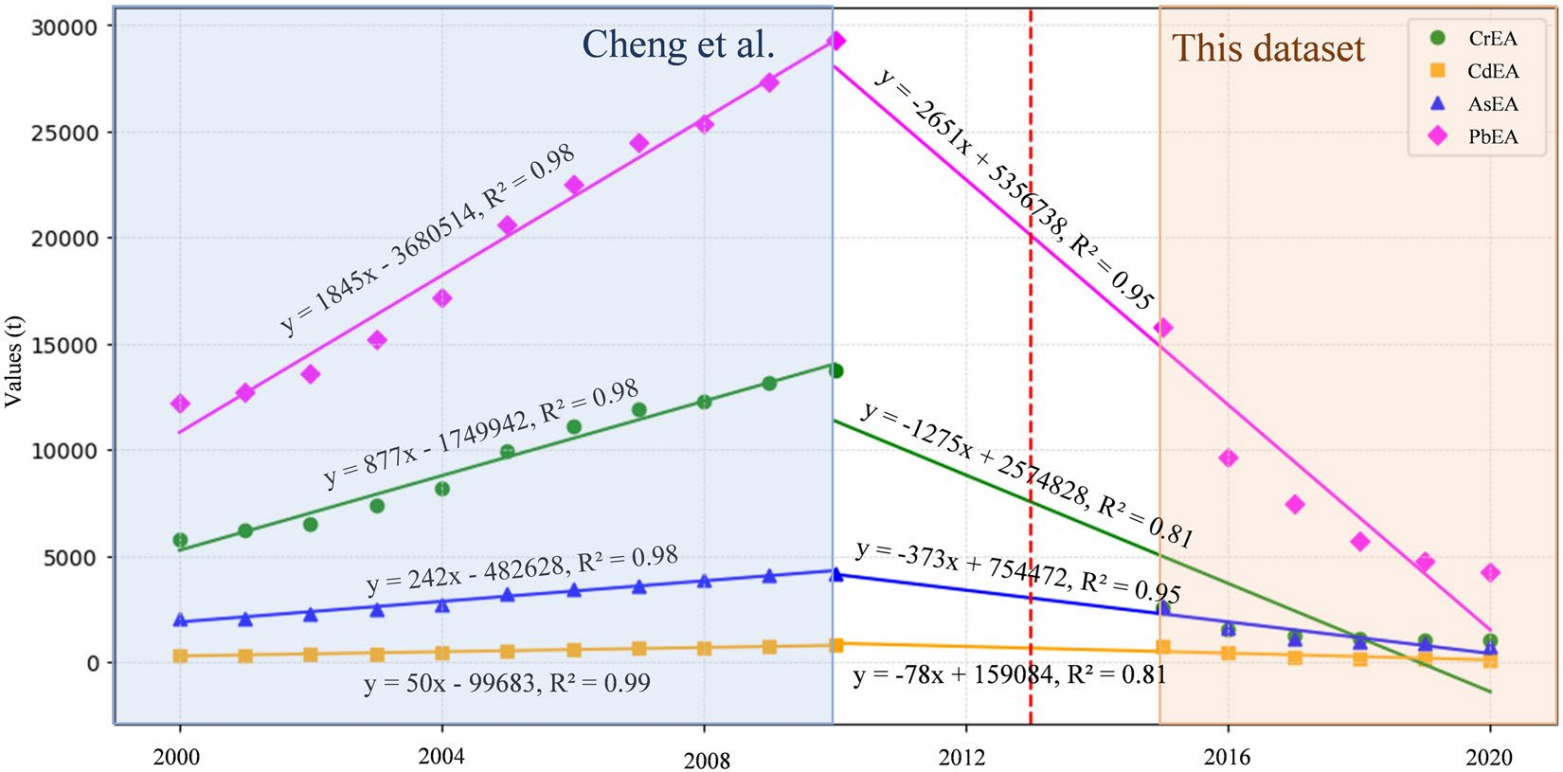
HMEAs emissions compared with other literature report. The red dashed vertical line represents the year 2013 when China’s Action Plan for the Prevention and Control of Air Pollution was implemented.

To further validate the data quality, we compared the predicted data with the reported annual emissions of four HMEAs by Cheng *et al*.^41^, as shown in Fig. 4. We performed linear regression between the 2010 data and our predicted data for the years 2015–2020, revealing a good fit with R^2^ values all exceeding 0.8. The R^2^ values for AsEA and PbEA were exceptionally high, exceeding 0.95. Due to the scale setting, CrEA and AsEA from 2015 to 2020 appear to overlap on the graph. This is because of the close proximity of their emission levels during these years. Detailed data can be found in the table below, with differences ranging between 4.7% and 15.5%, except for the year 2020, where the difference is 27.4%, resulting in the appearance of overlapping data points. However, in 2010, there is a substantial difference between the values of CrEA and AsEA (13715t and 4196t, respectively), leading to significant variations in the fitting R^2^. Notably, the data for the years 2011–2014 is currently unavailable. However, based on the graph, it is evident that after the implementation of China’s Action Plan for the Prevention and Control of Air Pollution in 2013, and following a year or two of preparation, there was a significant decrease in HMEAs by the year 2015. The policy’s effectiveness in implementation appears to be highly favorable.

### Model uncertainty

Regarding uncertainty, for a 95% confidence interval, the PICP values were 0.93, 0.94, 0.9, and 0.93 for CrEA, CdEA, AsEA, and PbEA, respectively. All four HMEAs’ PICP values are above 0.9, indicating that the data estimates are reasonable.

## Usage Notes

The results of this study have certain limitations due to the quality and quantity of data collected from published papers. The data distribution of some input features and output targets was inconsistent due to multiple variations in research objectives, methodologies, and experimental conditions. For instance, the HMEAs values were determined based on a wide range of features, including, but not limited to, emissions of household pollutants, vegetation coverage, municipal solid waste incineration, etc. Additionally, in this study, the dataset for HMEAs covers a 20-year span, while covariate data used for modeling are from 2015. This time difference may introduce errors. Future research should consider a time series approach to better capture the temporal variations in HMEAs.

Another significant concern involves the considerable disparities in the sources of different heavy metals across diverse regions. Employing a uniform contribution ratio for industrial sources could introduce uncertainty in estimation results. While acknowledging the validity of this concern, obtaining pollution source emission data categorized by industry for all 367 municipalities poses a formidable challenge. Addressing this issue is crucial for enhancing the model’s accuracy. In the event that detailed emission data by industry become available in the future, refining the model would be beneficial, presenting a potential focal point for subsequent research.

These constraints may cause uncertainties in some of the prediction results and may not precisely reflect real-world scenarios. Therefore, future research should focus on improving the ML model using a database that includes studies with well-defined scientific objectives and similar methodologies under uniform experimental conditions.

In addition, it is crucial to note that further research on the atmospheric emissions of other heavy metals, including mercury, copper, zinc, nickel, and so on, is essential. This extension of our study aims to contribute to a comprehensive understanding of the broader spectrum of atmospheric heavy metal pollutants and to support ongoing environmental research efforts.

## Data Availability

Data processing was performed in Python 3.10, and data used for the computation of HMEAs at city level are available can be accessed at Github repository located at https://github.com/Olivia-2012/HMEAs_DataSet. We implemented the procedure described in the Methods section.
